# Metabolic reprogramming of myeloid-derived suppressive cells

**DOI:** 10.18632/oncoscience.349

**Published:** 2017-04-28

**Authors:** Hong Du, Xinchun Ding, Cong Yan

**Affiliations:** Department of Pathology and Laboratory Medicine, Indianapolis, IN 46202, USA; IU Simon Cancer Center, Indiana University School of Medicine, Indianapolis, IN 46202, USA

**Keywords:** MDSCs, lysosomal acid lipase, mTOR, PPARgamma, cancer

Recent studies indicate that not only cancer cells go through metabolic reprogramming, immune cells also have distinct metabolic characteristics to influence their functions [1]. Myeloid-derived suppressor cells (MDSCs) critically contribute to tumorigenesis in three major ways. Firstly, MDSCs directly stimulate tumor cell proliferation, growth and metastasis; Secondly, MDSCs alter ECs' functions by influencing angiogenesis, tube formation, proliferation and permeability to facilitate MDSCs and cancer cell penetration across the vascular wall. Thirdly, MDSCs change organization of the thymus and spleen, impair progression of T cell development in the thymus, retard T cell maturation in the spleen, and weaken anti-tumor immune surveillance. These pathological functions are harnessed by the fatty acid metabolic pathway controlled by lysosomal acid lipase (LAL) [2–4].

Fatty acid metabolism is required for cell biosynthesis and bioenergetics of cell proliferation and survival. Mitochondrial fatty acid oxidation produces more than twice as much ATP per mole as oxidation of glucose or amino acids [5]. In fat catabolism, triglycerides and cholesteryl esters are hydrolyzed into fatty acids and cholesterol by LAL in lysosomes. Fatty acids are further broken down through a process known as beta oxidation that results in acetyl-CoA. Beta oxidation of fatty acids with an odd number of methylene bridges produces propionyl CoA, which is converted into succinyl-CoA and fed into the TCA cycle in the mitochondria. In the absence of the regular supply of fatty acids during LAL deficiency, the energy consumption is reprogrammed toward extracellular glucose consumption to fuel oxidative phosphorylation (OXPHOS). This was first observed by gene microarray analysis, in which expression of glycolytic metabolic genes was increased in LAL-deficient MDSCs [6]. Glycolysis breaks glucose (a six-carbon molecule) down into pyruvate (a three-carbon molecule). Pyruvate moves into mitochondria to be converted into acetyl-CoA by decarboxylation and enters the TCA cycle. In addition to glycolytic genes, gene expression of glucose transporters (GLUT3, GLUT6, GLUT8, GLUT12, and GLUT13) and the TCA cycle enzymes was also significantly increased in LAL-deficient MDSCs-like cells [7].

This metabolic reprogramming of energy consumption comes with penalties, very similar to cancer cells [6–8]. *1)* In LAL-deficient MDSCs, the mitochondrial membrane potential was impaired in association with increased expression of respiratory chain proteins (including NADH dehydrogenases, cytochrome proteins, ATPases and mitochondrial ribosomal proteins), increased glycolysis and TCA metabolic rates, and increased ATP production; *2)* LAL-deficient MDSCs showed increased expression of both lactate dehydrogenase A and B. In cancer cells, this altered metabolic process of aerobic glycolysis is advantageous to better survival; *3)* ROS concentration was significantly increased in LAL-deficient MDSCs, accompanied with up-regulation of the nitric oxide/ROS production genes, glutathione peroxidase/ glutathione reductase genes, and glucose 6-phosphate dehydrogenase gene. High level of ROS allows for the stimulation of cell proliferation, induction of genetic instability, and evasion.

This fatty acid to glucose metabolic reprogramming is controlled by overactivation of PI3K/ thymoma viral proto-oncogene (AKT)/mammalian target of rapamycin (mTOR), a master regulator of metabolic switch in cells. This was first revealed by Ingenuity Pathway Analysis in association with altered expression of other genes involving cell growth, cell cycle entry, cell survival, cell migration, histone epigenetics, and bioenergetic pathways [6]. Inhibition of mTOR, Raptor, Rictor, and Akt1 by pharmacological inhibitors or siRNA interference suppressed stimulation of tumor proliferation, progression, metastasis, and reduced immunosuppressive function of LAL-deficient MDSCs. These were concomitant with reversal of LAL-deficient MDSCs development from bone marrow Lin-progenitor cells, ROS overproduction, and impairment of mitochondrial membrane potential [2, 4].

As a control center of signaling for metabolic reprogramming [9], lysosome genesis and trafficking is regulated by Rab7 GTPase protein, which belongs to a superfamily of small-molecular-weight GTPase known to regulate intracellular membrane trafficking from early to late endosomes and lysosomes. A physical protein-protein interaction between Rab7 GTPase and mTOR, and colocalization on lysosomes of myeloid cells were observed [10]. Knocking down overexpressed Rab7 GTPase reversed the altered lysosome/mTOR distribution, reduced over-activation of mTOR, decreased glucose consumption, decreased ROS over-production, and increased healthy mitochondria. Inhibition of Rab7 GTPase led to reduced LAL-deficient MDSCs differentiation from bone marrow Lin-progenitor cells, reduced trans-endothelial migration, reversed immunosuppression and tumor stimulation [10].

Fatty acid metabolites serve as hormonal ligands for nuclear receptors, such as peroxisome proliferator-activated receptor gamma (PPARγ). PPRAγ ligand treatment of LAL-defi MDSCs was able to correct mTOR-associated malfunctions, and reversed immunosuppression and tumor stimulation [11]. When the PPARγ function is impaired by a dominant negative form of PPARγ (dnPPARγ) expressed in the myeloid-specifi cells of the c-fms-rtTA/(tetO)_7_-dnPPARγ bitransgenic mice, tumor growth and metastasis were enhanced. Bone marrow MDSCs isolated from these mice stimulated tumor cell proliferation and migration. Similar to LAL-defi MDSCs, MDSCs with dnPPARγ overexpression showed increased transendothelial migration, overactivation of the mTOR pathway, and ROS over-production [11]. These results indicate that PPARγ mediates neutral lipid metabolic signaling controlled by LAL, which provides a mechanistic basis for clinically targeting MDSCs to reduce the risk of cancer proliferation, growth and metastasis.

## References

[1] Biswas SK (2015). Immunity.

[2] Zhao T (2015). Oncogene.

[3] Zhao T (2014). J Immunol.

[4] Ding X (2014). Am J Pathol.

[5] Boroughs LK (2015). Nat Cell Biol.

[6] Yan C (2012). PLoS One.

[7] Ding X (2015). PLoS One.

[8] Qu P (2011). J Immunol.

[9] Settembre C (2013). Nat Rev Mol Cell Biol.

[10] Ding X (2017). Oncotarget.

[11] Zhao T (2016). Oncotarget.

